# Wireless sensor system for real-time performance monitoring in sports

**DOI:** 10.3389/fspor.2023.1305117

**Published:** 2023-11-27

**Authors:** Martin F. Berg, Henrik Døsvik, Kirsti Ø. Skjølsvik, Thea Solberg Pedersen, Viljar Aasan, Martin Steinert, Sindre W. Eikevåg

**Affiliations:** ^1^ Department of Mechanical and Industrial Engineering, Norwegian University of Science and Technology, Trondheim, Norway; ^2^ Beitostølen Healthsports Center, Innlandet, Norway; ^3^ Department of Civil and Environmental Engineering, Center for Sports Facilities and Technology, Norwegian University of Science and Technology, Trondheim, Norway

**Keywords:** sensor development, live performance monitoring, paralympic crosscountry sit-skiing, paralympic equipment, wireless sensors

## Abstract

In Paralympic sports, investigating seating ergonomics and optimizing for performance is crucial due to individual impairments. Usually, experiments are conducted in laboratory environments and for skiing, usually on a treadmill. In this paper, we are moving experiments out of the laboratory setting to in-slope performance monitoring of kinetics and kinematics. A wireless sensor system is developed and validated in terms of delay. The results show a median delay of 52 ms for the wired main system and 53 ms for the wireless sub-system. The sensor system was implemented on a highly adjustable Paralympic sit-ski, and an experiment was conducted to pinpoint optimal equipment settings for an individual athlete. In addition, the system provided force data from both knees, seat, belt, and both poles. The data collected can also be used to analyze the technique, in addition to assisting in the classification process in the LW10–12 class. The proposed system design also allows for adding a vast amount of different sensor types, and by testing for delay, synchronized with well-known GNSS and IMU sensors already used in many sports to analyze athlete performance.

## Introduction

In Paralympic sports, equipment significantly impacts athlete performance and injury prevention. Due to different impairment levels, equipment settings must be carefully investigated to ensure good work ergonomics and performance-enhancing motion envelopes. In Paralympic sit-skiing, the athletes utilize the upper body to generate propulsion by double poling. By using new sensor technology, we can analyze performed techniques and provide information to the coaches on how to gain improvements. Traditionally, athlete analysis is performed in a laboratory setting, but in-field skiing on the slopes can obtain an even better athlete insight. New sensor technology can also generate data on equipment modifications and can be used to tailor equipment to specific impairments. In Paralympic sports, many athletes use nonspecialized sit-skis, even in the Paralympics, without knowing if there is still room for improvement. As new technology has enabled many new sit-skies designs in recent years, we argue that by making information on optimizing for performance, using open-source technology and commercially available sensors, athletes can perform to their full potential, making the sports fairer for all athletes. Sensor systems can also be used for in-depth classifications, ensuring more fair competition within each Paralympic class. By using new sensor systems in paralympic skiing, we can allow the athletes to perform to their fullest potential, ensuring fair, exciting, and competitive sports.

In the competitive world of elite sports, athletes continuously try to gain an advantage over their opponents. A key factor contributing to success is optimizing training and technique through dedicated practice and analysis (1, 2). Even marginal improvements can significantly impact overall performance (3). The development of new sensor technology enables us to investigate those incremental improvements, and as the technology continues to evolve, our ability to analyze and comprehend them expands significantly. Consequently, employing advanced tools and methods to facilitate effective training and technique analysis becomes crucial. Sensors offer valuable means of quantifying an athlete's movements, which can be challenging to observe and measure with the naked eye, especially considering the quick movements in some sports. By capturing precise data on forces, accelerations, and other relevant metrics, coaches and athletes can make informed decisions regarding training adjustments, injury prevention, and performance optimization (4). Wireless connectivity for health and sports monitoring using Bluetooth and Zigbee to collect data unobtrusively without hindering movement has been proven to work great for measuring athlete performance (5). Combining multiple sensors in a wireless network would be beneficial, though a method of fusing multi-sensor data would be required. This can be achieved by using local clocks on sensor nodes or with the implementation of algorithms (6). A study of “Real-Time Athlete Monitoring” used small wireless sensor nodes to transmit player positions for soccer players to a base station, though it resulted in a system with an unacceptably high delay (7). Another study discusses several different applications where real-time streaming of sensor data would be beneficial (8). However, it concluded that no existing wireless technologies were able to satisfy the requirements of low delay and high bit rate.

The technology-driven approach enables a new era of informed training. Integrating sensors into existing equipment offers a way to bridge the gap between various off-the-shelf systems that lack communication. These previously isolated systems can exchange information and insight by embedding sensors and using compatible data protocols, unlocking new capabilities. By integrating sensors into equipment like rowing oars, tennis rackets, and ski poles, we can gain access to real-time data on their performance. Coaches and athletes can pinpoint areas of improvement. By measuring impact forces, equipment angles, and techniques, this data can be further analyzed to uncover patterns and areas of potential. The method of measuring performance depends significantly on which sport is analyzed. In a typical team sport, such as soccer, basketball, and handball, coaches and athletes have seen great effects of using motion tracking with cameras to analyze the positions of the players and the balls to develop new strategies (9–11). Cameras have also effectively studied the trunk movement and force curve of athletic paralympic rowing (12). For long-distance running blood, lactate measurement has proved to be a great tool for the prediction of performance and for adapting workout routines to improve efficiency (13, 14). For other sports, such as swimming, the measurement of speed using tachometers and time is commonly used to measure performance (15). An article evaluating ski sport dynamics used inertial measurement units (IMU), which can accurately measure 3D movements and acceleration (16–19), and load cells to obtain objective data from different ski sports (20). By combining objective force and acceleration measurements, it is possible to create a system that can accurately measure the real-time effort from athletes in watts (21).

GNSS (Global Navigation Satellite System) uses similar technology as GPS and requires four satellites to provide position and timing. This technology has become increasingly available for commercial use in Sports, making it possible to track distances and velocity during runs. Wearable sensors such as Admos (22) are designed for tracking athletes, using the GNSS data to analyze running performance. Systems like Protern (23) are using a Dish device connected to a video camera that syncs recorded video with a GPS time code. It uses GNSS to generate accurate linear timecode (LTC—audio time code) onto the audio track of the video, making it possible to sync data from their external Protern sensor. This is mainly used for Alpine skiing to analyze techniques and tactics (23).

Nordic skiing, they compete together in the class LW10–12 using a time penalty based on the different classes. In the classification process the athletes conducts various physical tests, measuring the activation of different muscle groups, providing a score that provides the classification of that athlete. One is called “the Board Test” that involves four different activities, where one includes the athlete sitting with their hands behind their neck and move their upper body 45 degree forward. Based on their performance to do this action, they get a score from 1 to 3. This test maps the lack of functionalities in trunk and hip, where LW12 has a score of 12 and LW10 is a score of 0–2. Putting an athlete in the right classification is crucial to ensure fair competition, and additional sensors may aid this process in the future (24).

This study introduces a versatile wireless sensor system designed for real-time performance monitoring in sports that can be synchronized to a system like Admos. The system is constructed from off-the-shelf components, ultimately creating a modular, open-source (25) sensor logging system. Furthermore, the system enables wireless, real-time monitoring and fusion of sensor data, much like the telemetry systems used in Formula 1 racing (26). By utilizing the system, it would be possible to research how different seating positions affect performance in cross-country sit-ski athletes, a subject that has been discussed in recent years (27–32). This system enables pinpointing of optimal technique for the individual athlete, creating more fairness in the sport.

## Method

**Figure 1:**
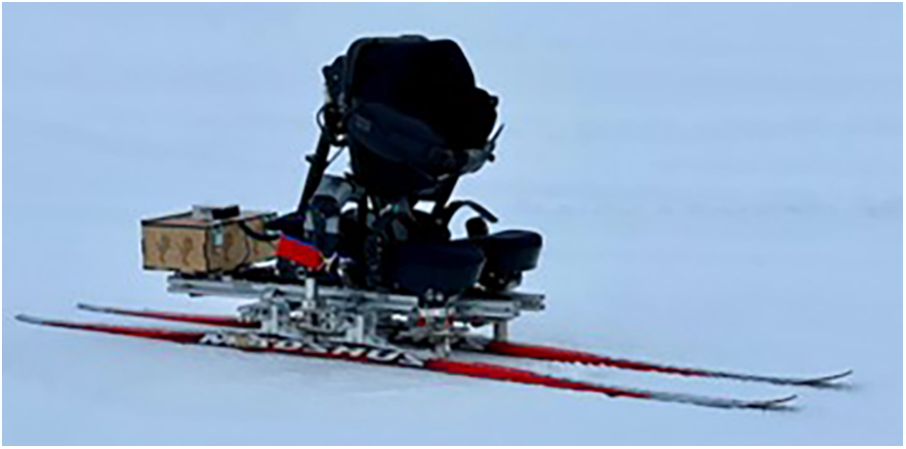
Fully adjustable sit-ski prototype with embedded sensors for measuring kinematics and kinetics in paralympic sit-skiing.

This section outlines the methodological approach employed in the design and implementation of the system for athlete performance monitoring in Paralympic cross-country sit-skiing. It provides a comprehensive overview of the system architecture, hardware components, software implementation, and system evaluation, ensuring a thorough understanding of the systems functionality and capabilities. The developed prototype for in-situ monitoring of Paralympic sit-skiing in the LW10–12 class is presented in Figure 1. The prototype is capable of a vast amount of adjustments for testing seating position effect on athlete performance as well as embedded with load-cells for analysis of technique and data generation for product development and monitoring of muscle activation.

### System architecture

**Figure 2:**
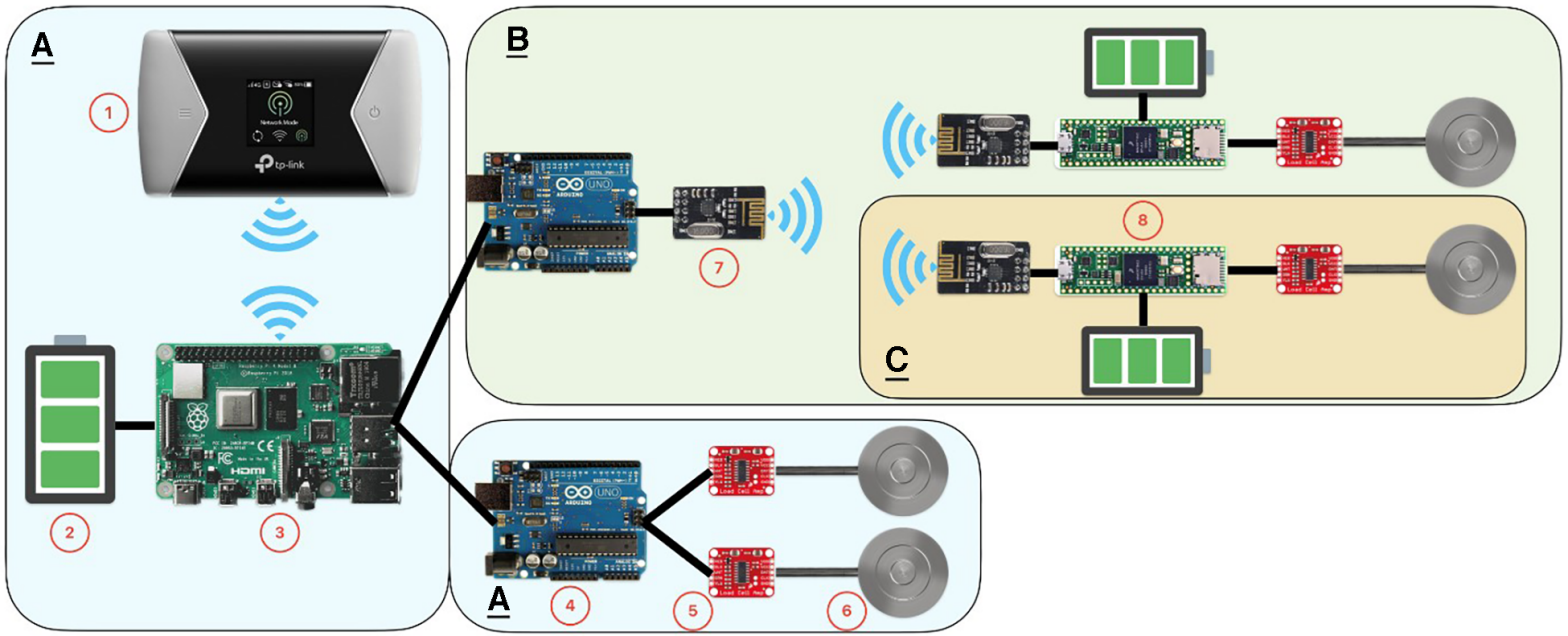
Hardware schematics. **A**: Main System, **B**: Sub System, C: Wireless Sensor Node. 1: TP-Link M7450 4G-Router, 2: Power Bank, 3: Raspberry Pi 4B 4 GB, 4: Arduino Uno R3, 5: HX711 Amplifier, 6: TAS606—Button Load Cell, 7: nRF24L01+ Wireless Transceiver Module, 8: Teensy 4.1 Microcontroller.

The proposed system, seen in Figure 2, consists of several off-the-shelf components, which are combined with software to enable the functions needed to provide data logging and real-time wireless data transmission.

**Figure 3:**
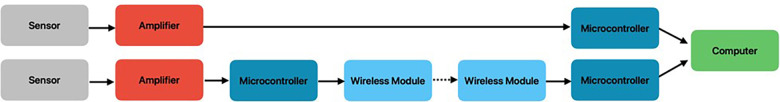
Flowchart of data signal.

The main system (Figure 2A) is a wired system using a USB protocol between the Arduino and Raspberry Pi. The signal originates from the load sensors, passing through several components until it is registered and logged by the computer, as illustrated in Figure 3. The TAS606—button load cell and TAS501—S-type load cell are analog load sensors that use a piezoresistive film to measure a load. The sub system (Figure 2B) is a wireless system, transferring sensor data between the wireless sensor node (WSN) (Figure 2C) and the main system.

This node can stream low-latency sensor data to the main system, which is placed on the sit-ski, where all the data is fused together. Through software and Wi-Fi, the main system is accessible wirelessly on any device with an internet connection, ultimately enabling live data transmission. All components and software solutions are interchangeable, making the proposed system a completely modular, open-source wireless sensor network.

### Hardware implementation

The following sections discuss the main system and sub-system configurations, highlighting the critical role each component plays in the system and ensuring accurate and real-time performance monitoring.

The system consists of a single board computer (SBC), microcontrollers, sensors, a 4G WIFI router, and powerbanks. The SBC serves as the central processing unit and data logger, while the microcontrollers manage the data acquisition from the sensors. The 4G Wi-Fi router enables wireless communication between the SBC and remote devices, facilitating real-time data transmission. The powerbanks are used to power all the components. In the following list, the components used in the development of this system are included.

• Raspberry Pi 4B 4 GB
• TP-Link M7450 4G-Router
• Arduino Uno R3 Microcontroller
• Teensy 4.1 Microcontroller
• TAS606—Button Load Cell
• TAS501—S-type Load Cell
• HX711 Amplifier
• nRF24L01+ Wireless Transceiver Module
• Powerbank 25,600 mAh
• Powerbank 3,350 mAh

The Raspberry Pi 4 Model B 4GB (Raspberry Pi Foundation, England) was chosen due to its performance and form factor. It is powered by a Broadcom BCM2711 quad-core Cortex-A72 (ARM v8) 64-bit SoC, running at 1.5 GHz, consuming a maximum of 1,280 mA, and comes with 4GB of LPDDR4-3200 SDRAM. The TP-Link M7450 wireless 4G router (TP-Link, China) was chosen due to its small size and fast, stable internet connection of 867 Mbps. In addition, it has a battery size of 3,000 mAh that provides a WiFi connection for 15 h continuously. The Arduino Uno R3 (Arduino, Italy) and Teensy 4.1 (PJRC, United States) microcontrollers were chosen due to their versatility and user-friendliness. The Arduino Uno has a powerdraw of 42 mA, while the Teensy has a maximum draw of 100 mA. To measure loads acting between the athlete and the equipment, the TAS606—button load cell (SparkFun Electronics, United States) is connected to the microcontroller. This is a single-point load cell capable of measuring forces up to 200 kg with a sensitivity of 1 mV/V. To use this loadcell effectively, an amplifier is needed to convert the small voltage changes into a signal that can be read by a microcontroller. The HX711 amplifier (Sparkfun Electronics, United States) was used. The amplifier is designed to work with low-voltage sensors and has a nominal power consumption of 1.6 mA. It incorporates a low-noise, programmable gain amplifier and a 24-bit analog-to-digital converter with an 80 Hz refresh rate. This is the limiting factory for data collection for the presented system architecture, therefore all data was collected at 80 Hz.

To enable low latency data transmission between the WSN and the main system, nRF24L01+ Wireless Transceiver Modules (HiLetGo, China) were used. This component is designed for low-cost and robust communication by using the 2.4 GHz ISM (The Industrial, Scientific, and Medical) band with high power efficiency only using 13 mA, where we can expect a latency of at least one millisecond (33). It can communicate with six modules at once with data rates up to 2 Mbps, allowing the system to receive data from up to five wireless sensor nodes simultaneously.

Connected, as Figure 2 illustrates, the main system has a maximum power consumption of 1,383.4 mA with two microcontrollers and four loadcells connected, calculated from the obtained values from each component. From an article analyzing the specified capacity in Power Banks, the theoretical output is 74% of the actual capacity (34). Combined with the powerbank of 25,600 mAh, the system can theoretically be used continuously for 13.6 h. The WSN has a maximum power consumption of 114.6 mA with one load cell attached. Combined with a powerbank of 3,350 mAh, the system can be used for 21.6 h with an efficiency of 74%.

### Software implementation

This chapter provides an overview of the software implementation for the wireless sensor system, encompassing the operating system, custom Python script, microcontroller firmware, and additional software to enable remote access. The software components play a vital role in ensuring seamless integration, data acquisition, and real-time monitoring of the system. All custom code can be found in the GitHub-Repository: “LoggingTool” (35).

On the central processing unit, the Raspberry 4B, Raspberry Pi Desktop OS was installed. To extract the data from the microcontrollers, a custom Python script was developed. The script enables the computer to read serial data from multiple USB ports simultaneously on the computer and append the data to a common CSV file. Additionally, the script automatically names the file with the current time the logging started, as well as it adds a timestamp with millisecond accuracy for every data-signal it registers. The collected raw sensor data from the sit-ski is processed through another Python script using the SciPy library, which is adjusted to identify the peak and valley of the forces, and normalize each double poling cycle from the athlete's movement. The number of datapoints varies for each cycle, as the frequency changes based on the slope and equipment condition. The script then plots the average value with a solid line and the 95% convergence area for each sensor with a shaded area.

The firmware for all microcontrollers was developed through Arduino IDE (Integrated Development Environment). On the main system, there is one microcontroller that receives data from locally connected load sensors, while another is dedicated to receiving data from the wireless sensor nodes. Additionally, the microcontrollers on each wireless sensor node have their own dedicated ID, so the data from each wireless sensor node can be identified.

### System evaluation and testing

To ensure the reliability and effectiveness of the wireless sensor system, a series of tests were conducted. These tests aimed to evaluate the system's performance in terms of delay, accuracy, and scalability. By investigating delay and adjusting timestamps accordingly, the presented sensor system is capable of sensor fusion with other systems where GPS timestamps are provided. The following subsections outline the testing methodologies for each of the tests performed.

### Delay

The first test assessed the delay from the moment the load was applied to the load cells until it was registered by the Raspberry Pi. This measurement is critical in determining the system's suitability for real-time monitoring applications. Figure 3 illustrates the path of the data signal.

**Figure 4:**
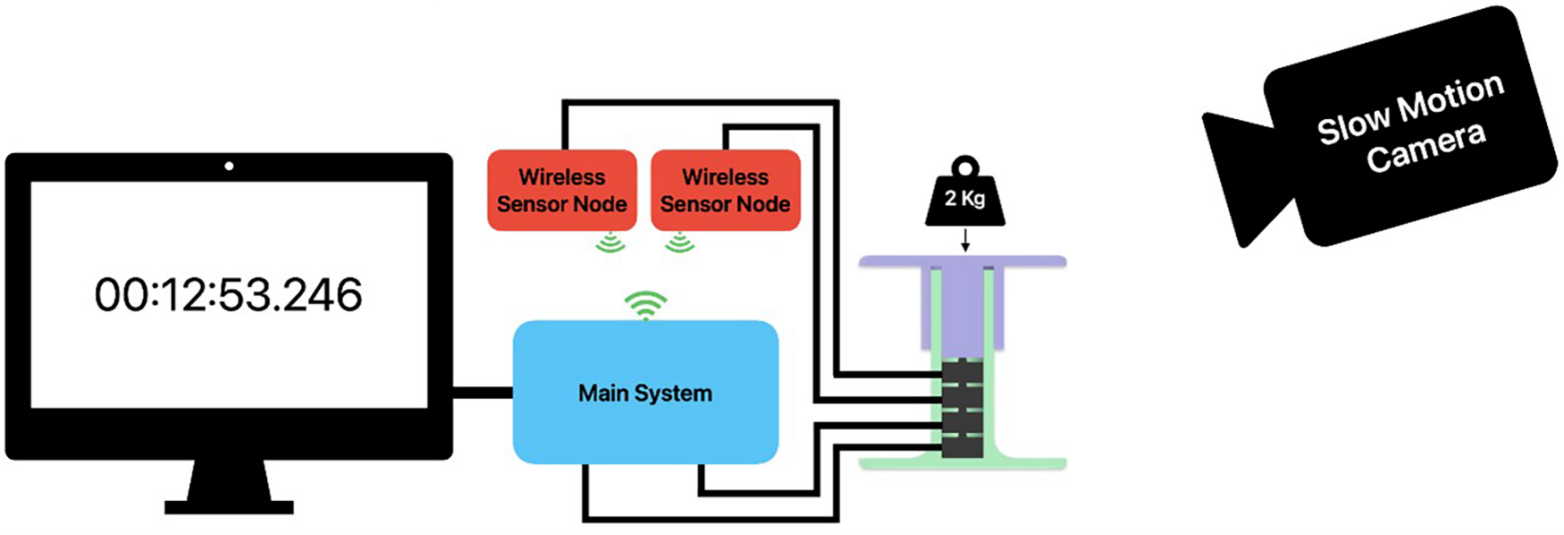
Test setup for delay measurement.

One button load cell was connected to the main system, and one to a wireless sensor node. To uncover each system latency, a window displaying the computer's internal system clock with milliseconds was presented on a 120 FPS monitor, providing an error margin of one frame equal to 8.33 ms, while a weight of 2 kg was dropped a few centimeters above the test jig. Both the test jig and the monitor were recorded using a 240 FPS slow-motion camera, providing an error margin of one frame equal to 4.17 ms. An illustration of the test setup can be viewed in Figure 4. To obtain the latency from touchdown until it is logged, the timestamp from the monitor was compared with the timestamps in the log file. The process was repeated ten times for each test to obtain an average delay value. The locally connected sensors and the wireless sensor nodes were tested separately to minimize sources of error.

The second test examined the system's scalability by evaluating any additional delays or performance degradation when incorporating more, or different, components to the setup. This aspect is crucial in determining the system's adaptability to various applications that may require more sensors, as well as its modularity by using other hardware components. Firstly, one by one sensor was added locally to each system to test the scalability. To ensure that all sensors are activated simultaneously with the same force, a 3D-printed test jig was created. This fixture stacks all the sensors on top of each other, ensuring that the applied force is uniformly distributed on all load cells simultaneously. Secondly, multiple WSNs were tested. Thirdly, a Teensy 4.1 microcontroller was tested on the sub system to test the modularity.

### Implementing the sensor system in a paralympic Sit-Ski to improve athlete performance

**Figure 5:**
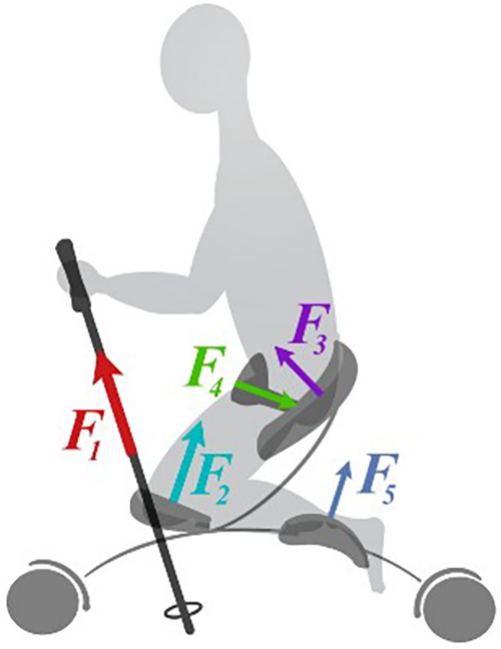
Forces acting between a sit-skier and his equipment.

The system was tested on a male paralympic sit-skier in the LW-12 class, age 25, at Beitostølen, Norway. The athlete is a person of short stature, approved by the Norwegian Centre for research Data (ID 514085), and provided informed written consent prior to the study. The athlete had an adjustable sit-ski manufactured by Skeno, Norway, modified specific to his body. The system was strategically placed on the athlete's skiing equipment so that the forces acting between the athlete and his equipment, illustrated in Figure 5, could be measured during a test run, to get a better understanding of the sport and how different seating positions can affect performance. A total of six load cells were connected to the system, four on the main system mounted on the sit-ski itself, and two load cells connected to two WSNs, used to measure forces in the ski-poles from the athlete. The force sensors on the knee and foot were placed anterior to the leg, meaning they got compressed based on the force distribution during skiing, while the sensor on the seat and belt was placed posterior of the body. The loadcell on the seat was compressed, and the belt sensor got stretched during runs. The athlete tested four different seating positions were the aggressive position with knees lower than the ankles and more upraised upper body had a major reduction in time compared to the position showed in Figure 5. This position is served as the baseline for this paper.

**Figure 6:**
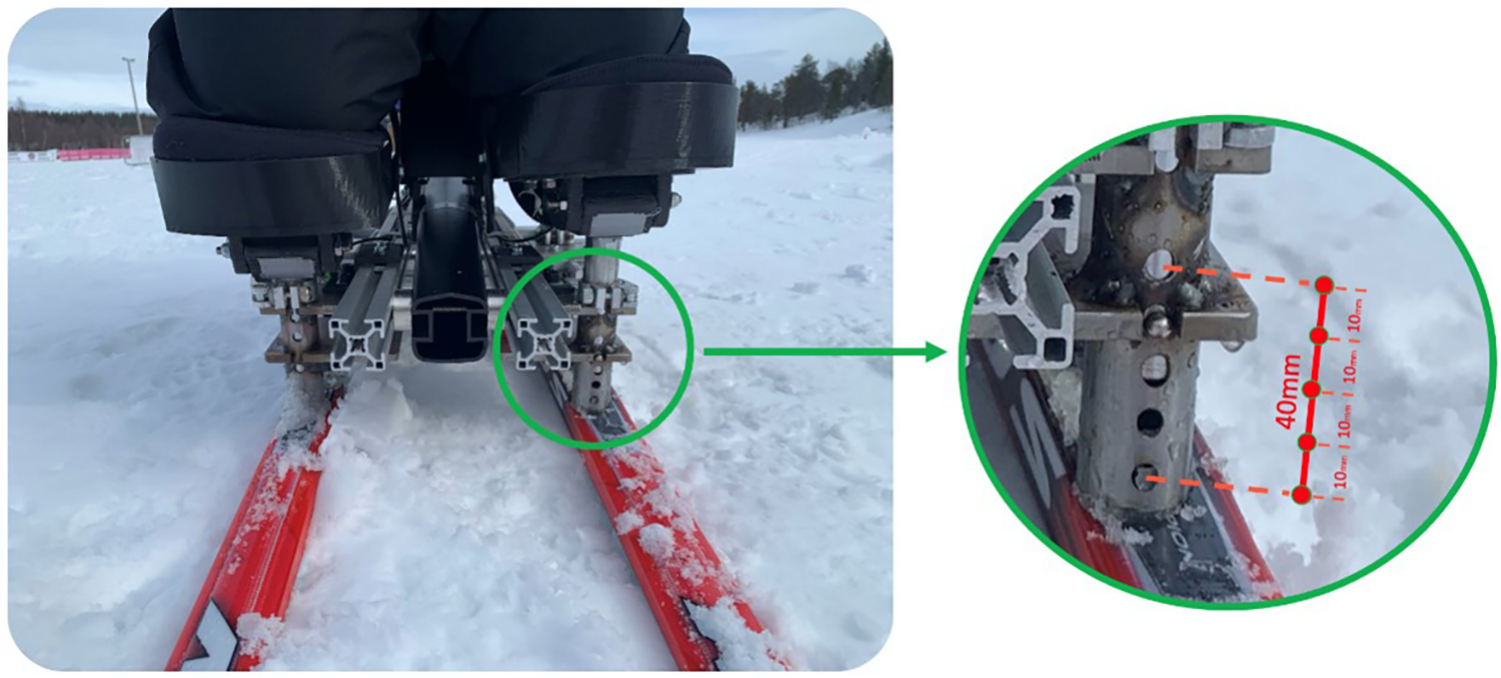
Altering knee height by adjusting equipment.

Due to the medical condition, the athlete had different leg lengths. An additional experiment was conducted to leverage the sensor system´s capabilities in identifying any asymmetry and determining the optimal adjustment by altering the height of the knees, see Figure 6. The athlete was instructed to go through the test slope multiple times while one knee was elevated by 10 mm for each run. The goal was to continue adjusting the height until the optimal level was achieved by analyzing the data.

**Figure 7:**
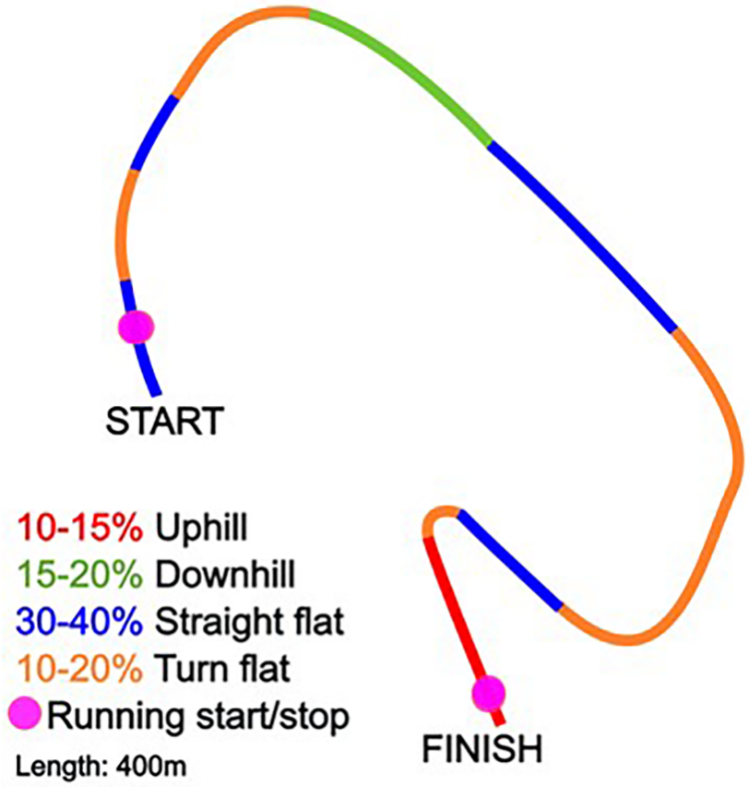
Outline of the track used during testing.

The tests were executed at Beitostølen Helsesportsenter (Innlandet, Norway). The tests were conducted during sunny conditions with temperatures approximately −5 degrees Celsius, with minimal changes in snow condition. This is a 400 m track designed to challenge the athlete on technique, consisting of uphill, downhill, flat turn, and straight flats as visualized in Figure 7. Before testing the athlete did some test runs to familiarize themselves with the track. The slope was according to the paralympic regulations (36).

## Results

In this chapter, we present the results obtained from the delay and scalability test, as well as the results from the practical implementation of the wireless sensor system for real-time performance monitoring in athletes. All individual tests were performed ten times to obtain an average value.

### Delay test results

**Figure 8:**
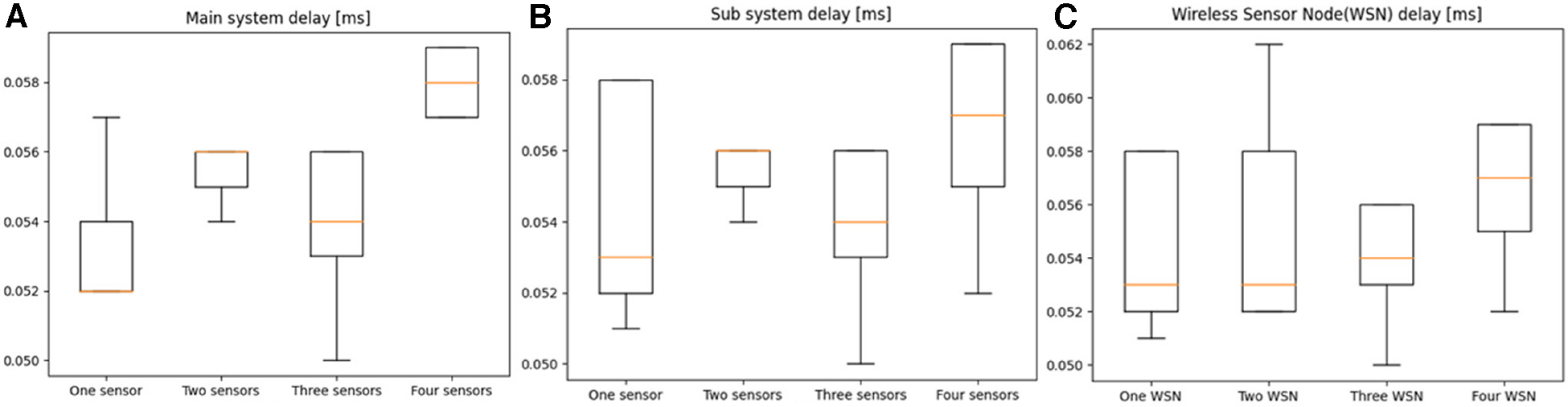
Delay result from (**A**) main system, (**B**) Sub system and (**C**) wireless sensor nodes (WSN).

In Figure 8A, a comparison is presented between the measured delays of the main system with varying numbers of attached sensors (ranging from one to four). The median delay was found to be 52 ms with a singular sensor. As more sensors were incrementally added, the delay increased to 58 ms. Interestingly, the minimum delay varied by two milliseconds for all sensors, with a range of 50–57 ms. However, the maximum delay increased from 57 to 59 ms when four sensors were connected as compared to just one. Figure 8B displays the measured delays of the sub-system, which were tested with one to four sensors, mirroring the configuration of the main system. The average delay with one sensor connected was found to be 53 ms, only one millisecond more than that of the main system. With the gradual addition of sensors, the average delay increased to 57 ms. The minimum delay varied only by two milliseconds, with a range of 51–53 ms. However, the maximum delay increased from 58 to 59 ms when four sensors were connected compared to one. In Figure 8C, the outcomes of incorporating multiple wireless sensor nodes into the sub system are displayed, each containing a single sensor.

**Figure 9:**
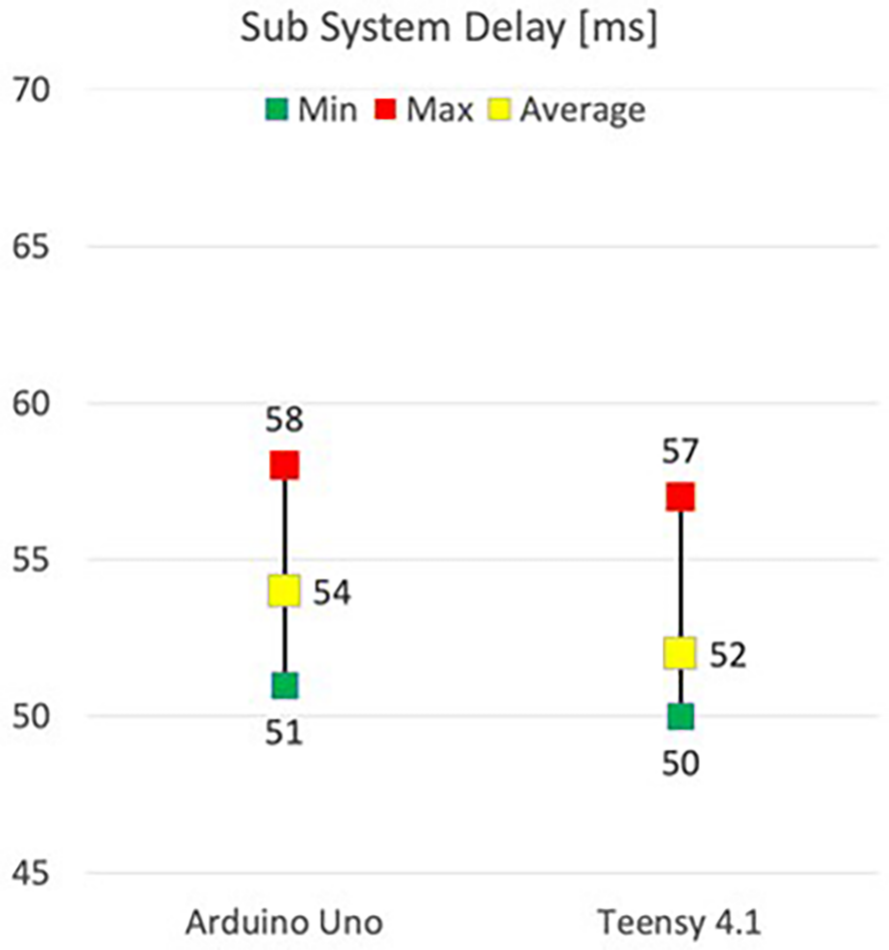
Minimum, average and Maximum delay for different microcontrollers.

The average delay for the sub-system with one WSN was 53 ms, gradually increasing to 57 ms with four WSNs. The minimum delay ranged between 50 and 51 ms. In contrast, the maximum delay varied from 58 to 62 ms, representing a slight increase when compared to previous results. Figure 9 showcases the results obtained from testing delay with different components.

When different microcontrollers for the WSN in the sub-system were tested, it was discovered that the Arduino Uno had a higher average delay of 54 ms, compared to the Teensy 4.1 with an average delay of 52 ms. Furthermore, the Teensy recorded a lower minimum delay of 50 ms compared to 51 ms for the Arduino. Finally, the maximum value was 57 for the Teensy, compared to 58 ms for the Arduino.

### Practical implementation results

**Figure 10:**
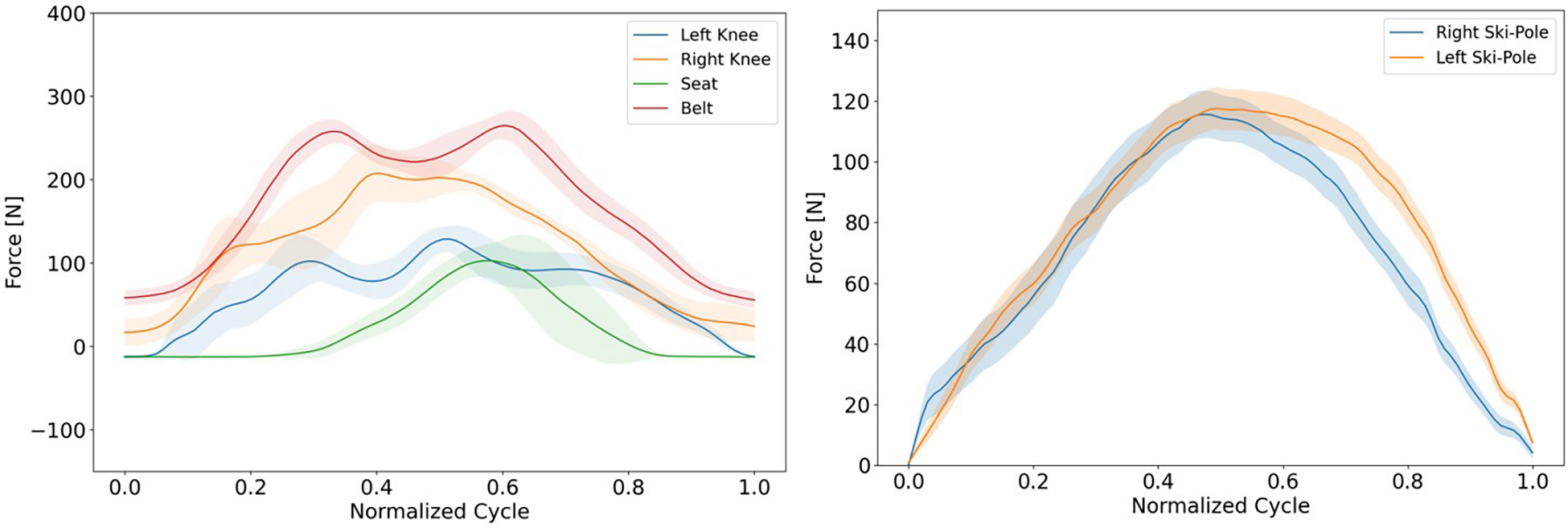
Processed example data from testing.

Figure 10 displays data gathered with the introduced sensor system during the practical implementation test at Beitostølen, Norway. The figure presents a comparison of the cycle data from both knees, seat, and the belt, with a 95% convergence area for each sensor. The results show a difference in the values registered for each knee, with a significantly higher force being detected by the sensor for the right knee. Some possible explanations for the observed asymmetry in the data could be that the athlete is stronger on one side of their body, or it may be due to a physical difference in the length of the athlete's limbs. Moreover, the seat sensor recorded low values, indicating that the athlete was leaning forward. This observation is supported by the high value registered by the belt sensor.

**Figure 11:**
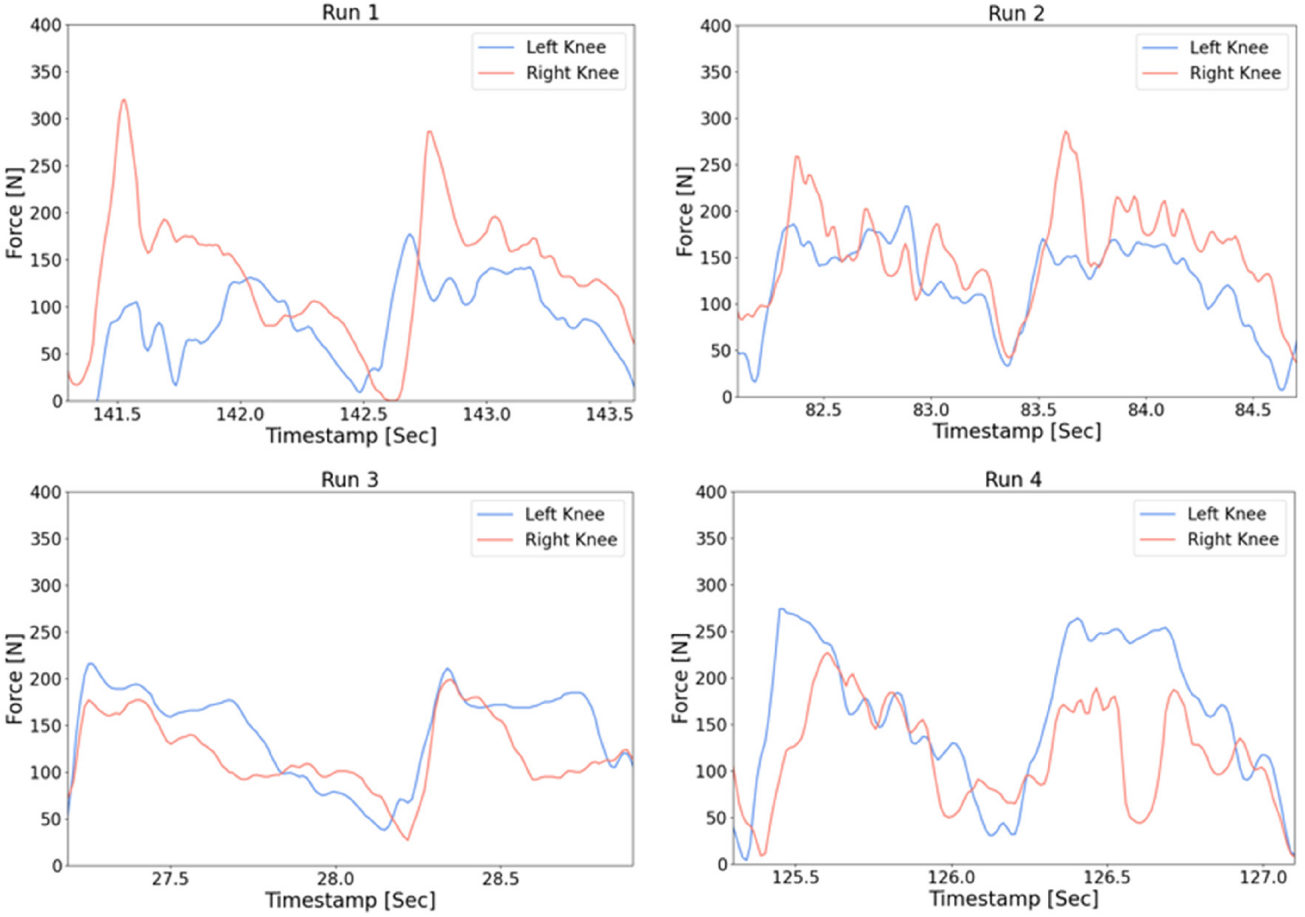
Raw data of knee forces from four runs.

Figure 10 presents data obtained from the wireless sensor nodes attached to the athlete's ski poles, which measured the force applied by the athlete to generate forward propulsion. The same script used to process the data in previous tests was also applied here. The results reveal that, similarly to the knee data, more force was applied to one pole than the other. In this case, the left pole registered a higher force, which contrasts with the knee data. In Figure 11, the raw sensor data from each knee is displayed, which was collected during the experiment aimed at determining the optimal adjustment of knee height to compensate for the athlete's different leg lengths.

**Figure 12:**
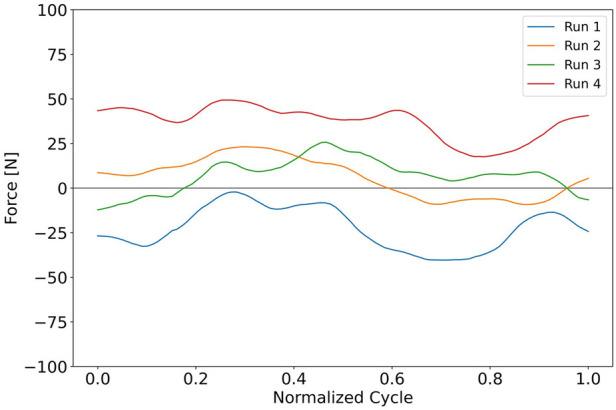
Processed data, the average difference in knees.

In “Run 1”, it is evident that the athlete is bearing more weight on their right knee compared to their left. By “Run 3”, the knees appear to be more evenly loaded, with a slightly higher value recorded in the left knee, indicating that the knee may have been raised excessively. Figure 12 displays the cycle data obtained from the knee sensors for each run, which were compared against each other.

As seen in the raw data from Figure 11, the average difference in the knees during “run 1” and “run 4” is significant, while for “Run 2” and “Run 3”, the difference is smaller. Interestingly, the value from “Run 2” suggests that the average force on the left knee is slightly higher than that of the right in the first section of a cycle.

## Discussion

In this study, we presented a versatile, modular, and open-source wireless sensor system for real-time performance monitoring in sports. The system demonstrated sufficient performance in terms of accuracy, reliability, and ease of use, providing valuable insights into athletes’ performance. Our findings suggest that the system efficiently captures precise measurements while remaining user-friendly, enabling coaches and athletes to effectively monitor and analyze data.

This system shows that it can be used for classification by measuring the different loads applied on the sit-ski, making it possible to measure the level of impairment the athlete has. By analyzing further the data gathered by the system, athletes and coaches can optimize both equipment an technique. The results from all tests are discussed in depth, emphasizing the system's efficacy and its potential for broader applications in the sports performance domain.

From the graphs in Figure 10, we can utilize this system to make a more qualified classification placement of athletes based on their force distribution and generated force on the equipment. By analyzing how much force an athlete generates in the different limbs, there is a possibility to differ between different level of impairment and verify that athletes are in the right class.

When comparing LW 10 to LW 12 class, there is a huge gap in trunk control. If we can detect higher generated force in that area, we can presume a higher level of activation in that muscle area. We are not proposing that the classification system should be changed, but the tools can aid the classification process, resulting in a fairer placement of athletes in the different classes and create a fairer competitive sport.

This tool can aid the athlete to perform to the best of their ability without the worries of the equipment slowing them down. The data provides the opportunity to optimize their equipment on an individual basis. By adjusting different parameters of the sit-ski, we can balance the force in the direction that is optimal for the athlete, increasing efficiency, but also preventing injury from extensive training and competition. We can also observe from Figure 10 that there is asymmetry in the force distribution on the right and left pole. The left pole generates more force in a slightly longer time, indicating that the athlete is stronger or leaning more on the left than right arm. This shows that we can both increase efficiency by optimizing equipment, but also increase efficiency in the double poling technique.

The case from Figure 11 shows how we can adjust the equipment based on the forces generated on each of the knees of the athlete. By observing the asymmetry of the forces, we can adjust and iterate the equipment to compensate and balance for symmetry. This is shown in Figure 12 where the average of the knees gets closer to zero in run 2. This may also prevent injuries as there is an equal load on the spine when skiing.

The delay test results indicated that our system is well-suited for real-time monitoring applications, exhibiting an average delay of 53 ms with one sensor on the main system and 54 ms for the sub system. By examining the delay of each component in the system, we can obtain valuable information about the efficiency of each component.

When the signal reaches the computer, it passes through various components before it is logged. By subtracting the average delay with the initial delay from the computer screen of 8.33 ms, the slow-motion camera of 4.17 ms, and the load amplifier of 12.5 ms, we obtain an estimated delay of approximately 28 ms. It is not possible to measure the delay in each component of the computer accurately, but it is assumed that some of it originates from the USB-controller and the driver used to communicate with the microcontroller (37).

The processing speed of the microcontroller depends on the interfaces used for communication with other components. Where the range can vary from 400 kHz to 8 MHz. By receiving and sending data we have a maximum delay of 5 microseconds based on the slowest speed, which is negligible. However, adding multiple sensors to a single microcontroller increases the delay by 2–3 ms for each sensor, likely due to simultaneous data signal processing.

For the subsystem, it was anticipated that the signal would experience an additional delay as it passes through more components while being wirelessly transmitted from one microcontroller to another through nRF24L01 + modules. However, it was observed that there was no measurable additional delay when employing the wireless sensor nodes, and the delay was nearly identical to the sensors connected to the main system when comparing in Figure 8. This is crucial, as it proves that time correction is unnecessary when fusing data from multiple sensors for analysis. It is essential to note that the wireless sensor nodes were situated close to the receiving module during testing, approximately 50 cm, and we can anticipate the delay to increase with greater distance.

During the system evaluation tests, some issues were encountered. When logging data from multiple microcontrollers simultaneously, there were some problems with merging the data, as the data from one microcontroller was sometimes delayed. Although it worked on occasion, it was not reliable, and it was decided to run two separate log scripts during the experiments at Beitostølen, Norway. Additionally, there were some complications with data logging, as the values from one specific sensor occasionally spiked from a normal range of 0 N and 1000 N to values between −600 N and +3,000 N. This was easily filtered out during post-processing. We believe this issue stems from either a faulty amplifier or loose wiring, as most connections in our test setup used clamps and not soldered connections.

The wireless sensor system's performance, as demonstrated by the conducted tests, confirmed its suitability for real-time monitoring and accurate measurement of athletes. The minimal delay, high accuracy, and scalability of the system showcase its potential for various sports performance monitoring applications. Future research will explore the integration of additional sensor types and the application of the system in different sports and contexts with the same low delay of 50 ms.

Future work on the system should include improvements in the custom python script that would enable it to reliably read data from multiple microcontrollers simultaneously and merge the data. Additionally, it could explore the integration of additional sensors, such as accelerometers and gyroscopes, to provide a more comprehensive view of athlete performance. Different communication protocols should be tested for decreasing delay. Using SPI or I2C for data transfer may decrease delay. As of now, the clock of each node and subsystem was not calibrated. This may introduce uncertainties on the delay between the system and should be addressed in a later iteration. Additionally, testing the system on a larger group of athletes, and in various sports, would help to further validate its effectiveness and versatility. Beyond sports, the modular design of our system could be adapted for use in healthcare, rehabilitation, or industrial settings where real-time monitoring is essential. However, in Paralympic sit-skiing, the presented system has proven to provide live in-field data in regard to kinetics and kinematics, which would greatly benefit the classification process and provide data for individual equipment development and analysis of technique.

## Conclusion

This paper presented a versatile, modular, and open-source wireless sensor system for real-time athlete performance monitoring. The system's modular design, built from off-the-shelf components, enables more people to use a wireless sensor system with real-time data monitoring. The system demonstrated sufficient performance in terms of accuracy, reliability, and user-friendliness, providing valuable insights into an athletes' performance in paralympic cross-country skiing. Through validation tests, the median delay was 52 ms for the main system and 53 for the sub system. However, these values will vary depending on the components used. Moreover, the potential of this system extends beyond the realm of sports, with possible applications in healthcare, rehabilitation, or industrial settings where wireless real-time monitoring of sensors is beneficial.

## Data Availability

The raw data supporting the conclusions of this article will be made available by the authors, without undue reservation.
